# The Histone Variant His2Av is Required for Adult Stem Cell Maintenance in the *Drosophila* Testis

**DOI:** 10.1371/journal.pgen.1003903

**Published:** 2013-11-07

**Authors:** Jose Rafael Morillo Prado, Shrividhya Srinivasan, Margaret T. Fuller

**Affiliations:** 1 Department of Developmental Biology, Stanford University, School of Medicine Stanford, California, United States of America; 2 Department of Genetics, Stanford University, School of Medicine, Stanford, California, United States of America; University of Cambridge, United Kingdom

## Abstract

Many tissues are sustained by adult stem cells, which replace lost cells by differentiation and maintain their own population through self-renewal. The mechanisms through which adult stem cells maintain their identity are thus important for tissue homeostasis and repair throughout life. Here, we show that a histone variant, His2Av, is required cell autonomously for maintenance of germline and cyst stem cells in the *Drosophila* testis. The ATP-dependent chromatin-remodeling factor Domino is also required in this tissue for adult stem cell maintenance possibly by regulating the incorporation of His2Av into chromatin. Interestingly, although expression of His2Av was ubiquitous, its function was dispensable for germline and cyst cell differentiation, suggesting a specific role for this non-canonical histone in maintaining the stem cell state in these lineages.

## Author Summary

Many tissues in the body are maintained by adult stem cells, which are dedicated but undifferentiated precursors that both maintain their population throughout life and produce daughter cells that differentiate to replace cells lost to turnover or damage. Here we show that the histone variant His2Av is required cell autonomously for maintenance of both germline and somatic cyst stem cells in the *Drosophila* testis. Although His2Av is expressed ubiquitously, under normal conditions, function of this histone variant was not required for correct differentiation of stem cell progeny in testes or for the survival of cells in the developing eye. We propose that adult stem cells maintain a plastic, bipotential state able to switch between self-renewal and differentiation and that His2Av may provide a chromatin state that helps bias transcription programs towards the stem cell fate.

## Introduction

Many adult tissues with short-lived, highly differentiated cells such as blood and skin replace cells lost to turnover through the proliferation and differentiation of adult stem cells. Adult stem cells must also self-renew to maintain a source of differentiating cells in the long term. The mechanisms that control the balance between self-renewal and differentiation need to be tightly regulated to maintain homeostasis of adult tissues. Although recent work has focused on signals from the local microenvironment of the stem cell niche, responses to these signals take place in the context of cell autonomous properties of the stem cell state that influence the ability of adult stem cells to maintain their identity. Likely candidates for such cell autonomous properties include the state of chromatin at key regulatory genes that influence stem cell maintenance.

The basic unit of eukaryotic chromatin, the nucleosome, is formed by DNA wrapped around an octamer containing two copies each of histones H2A, H2B, H3, and H4. Access to DNA by transcription factors and RNA polymerase is achieved by factors that control the post-translational modifications of core histones [1] and/or remodel nucleosomes [2]. The replacement of canonical histones with histone variants has recently emerged as an additional mechanism regulating chromatin accessibility [3]. Variants of the canonical histone H2A are highly conserved across species and play roles in transcriptional control, formation of heterochromatin boundaries, lineage commitment, and DNA repair. In yeast and mammals, H2AX is involved in recruiting factors to the sites of DNA damage [4] and H2A.Z is implicated in transcriptional regulation [5], [6]. In *Drosophila*, His2Av, the only known variant of H2A, assumes functions of both H2AX and H2A.Z [7]. *Drosophila* His2Av and His2A share 55% of their amino acid sequences, with the C-terminal region of His2Av considerably longer than that of His2A [8].

Here, we show that the histone variant His2Av is required cell autonomously for the maintenance of two adult stem cell populations in the *Drosophila* testis. The stem cell-niche microenvironment at the apical tip of the *Drosophila* testis consists of the germline stem cells (GSCs), which give rise to sperm [9]; the cyst stem cells (CySCs), which give rise to the cyst cells that enclose germ cells as they differentiate [10], [11]; and the post-mitotic somatic hub cells, to which GSCs and CySCs attach [12], [13]. His2Av function is required for both GSC and CySC maintenance; however, its function was dispensable for the differentiation program in the germ and cyst cell lineages. Our results suggest that in the absence of DNA damaging agents, the transcriptional role of His2Av may be required to regulate the delicate balance between self-renewal and differentiation states in adult stem cells.

## Results

### *His2Av* is required cell autonomously for GSC maintenance

**Figure 1:**
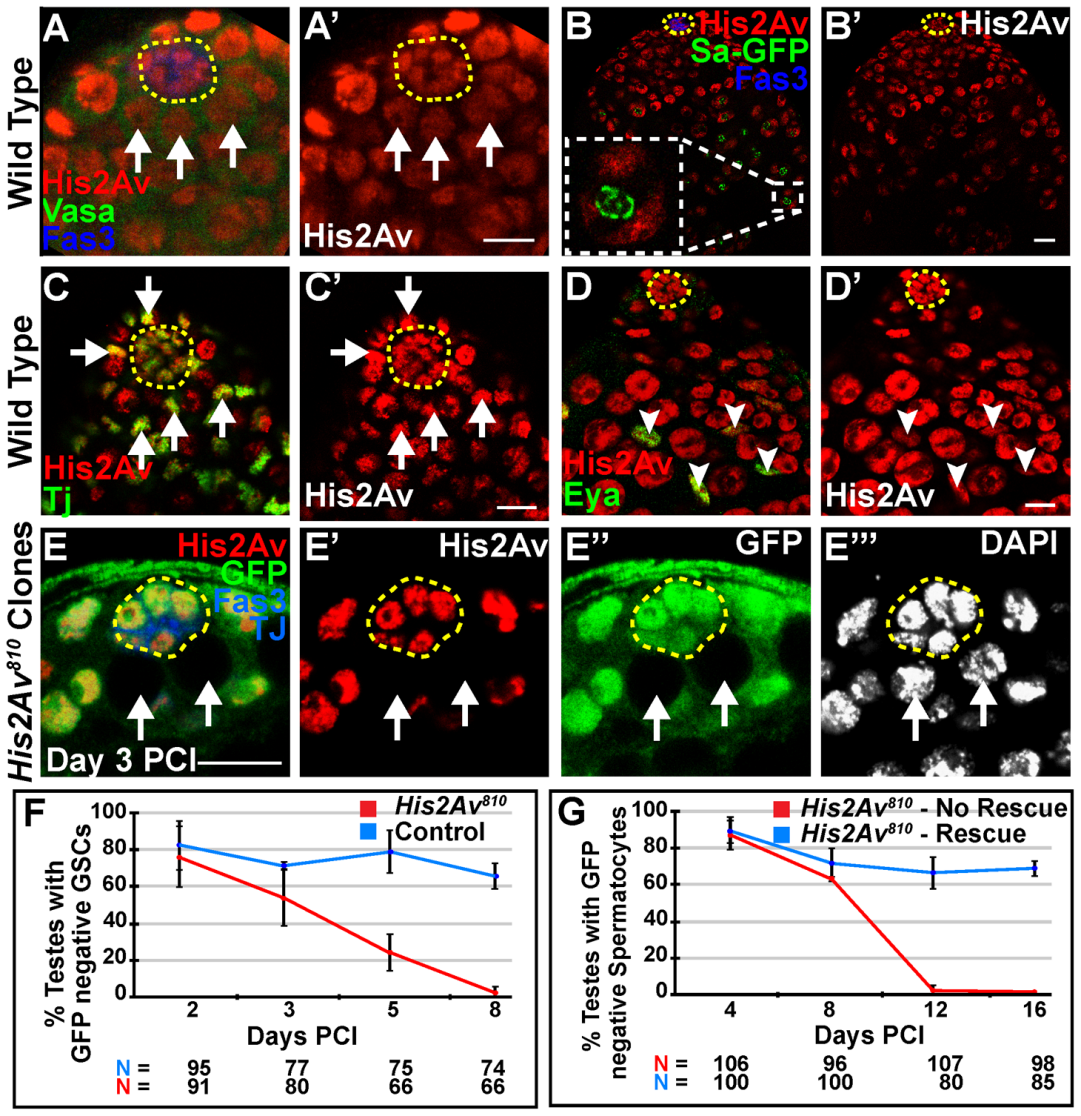
*His2Av* is required cell autonomously for GSC maintenance. (**A–A′**): Apical tip of wild-type testis immunostained with anti-His2Av (red), anti-Vasa (green), and anti-Fas3 (blue). GSCs (arrows) identified as Vasa positive cells adjacent to the hub (yellow dashed line). (**B–B′**): Testes expressing Sa-GFP (green) to mark spermatocytes, immunostained with anti-His2Av (red), anti-GFP (green), and anti-Fas3 (blue). Inset shows spermatocyte nucleus with Sa-GFP marking the nucleolus and His2Av localized to the chromosomes; hub (yellow dashed line). (**C–D′**): Apical tip of wild-type testes immunostained with anti-His2Av (red, C–D′), anti-Tj (green, C) to mark CySCs (arrows, C, C′) adjacent to the hub (yellow dashed line) or anti-Eya (green, D) to mark cyst cells (arrowheads, D, D′). (**E–E′″**): Testes day 3 PCI immunostained with anti-GFP (green) to identify homozygous *His2Av^810^* mutant GSCs (arrows), anti-His2Av (red), anti- Fas3 and Tj (blue) and DAPI (E′″); hub (yellow dashed line). Scale bars: 10 µm (**F**): Percentage of testes with *His2Av^810^* mutant (red) or FRT 82B control (blue) GSCs scored at indicated times PCI.(**G**): Percentage of testes with *His2Av^810^* mutant spermatocyte cysts in a genetic background either with (blue line, rescue) or lacking (red line, no rescue) a *His2Av-mRFP* genomic rescue transgene. Data shows average ± S.D.

Immunostaining of wild-type adult testes revealed His2Av protein expression in many cell types in the adult testis of *Drosophila*. At the apical tip of the testis, His2Av localized to the nuclei of somatic cells of the hub, GSCs (Fig. 1A) and CySCs (Fig. 1C). In differentiating spermatocytes, His2Av was concentrated on the autosomal and sex bivalent chromosomes within the nucleus (Fig. 1B, inset). His2Av also localized to the nuclei of differentiating somatic cyst cells associated with spermatocyte cysts (Fig. 1D).

Clonal analysis revealed that His2Av function is required cell autonomously for stem cell maintenance in the *Drosophila* male germline. Negatively marked GSCs lacking His2Av function were generated in adult fly testes by mitotic recombination using the FLP/FRT system in a *His2Av^810^*/+ background [14]. GSCs at day 3 post clonal induction (PCI) (Fig. 1E) and later germline clones at day 8 PCI (Fig. S1A) homozygous mutant for *His2Av^810^* did not exhibit His2Av staining, indicating specificity of the antibody towards His2Av protein and a sharp decline in protein levels in *His2Av* mutant GSCs by at least day 3 PCI. At day 2 PCI, GSCs homozygous mutant for *His2Av^810^* were detected in 75% of the testes scored, similar to the 81.4% observed in controls (Fig. 1F). By day 8 PCI, the percentage of testes with at least one marked GSC clone dropped to 2% for the *His2Av^810^* mutant (Fig. 1F), suggesting a defect in GSC maintenance upon loss of *His2Av* function, while 64.8% of control testes had at least one marked GSC.

Consistent with the loss of mutant GSCs, *His2Av^810^* mutant spermatocytes were not maintained over time after clone induction. At day 4 PCI, *His2Av^810^* spermatocytes were observed in 86% of testes. However, by day 12 PCI, *His2Av^810^* mutant spermatocytes were no longer observed (Fig. 1G). A genomic transgene carrying the *His2Av* coding sequence under control of its endogenous promoter and fused to the mRFP coding sequence (*His2Av-mRFP*) [15] rescued the loss of spermatocytes, indicating that the failure to maintain GSCs and their differentiating progeny was due to loss of His2Av function (Fig. 1G, Fig. S1B, C).

**Figure 2:**
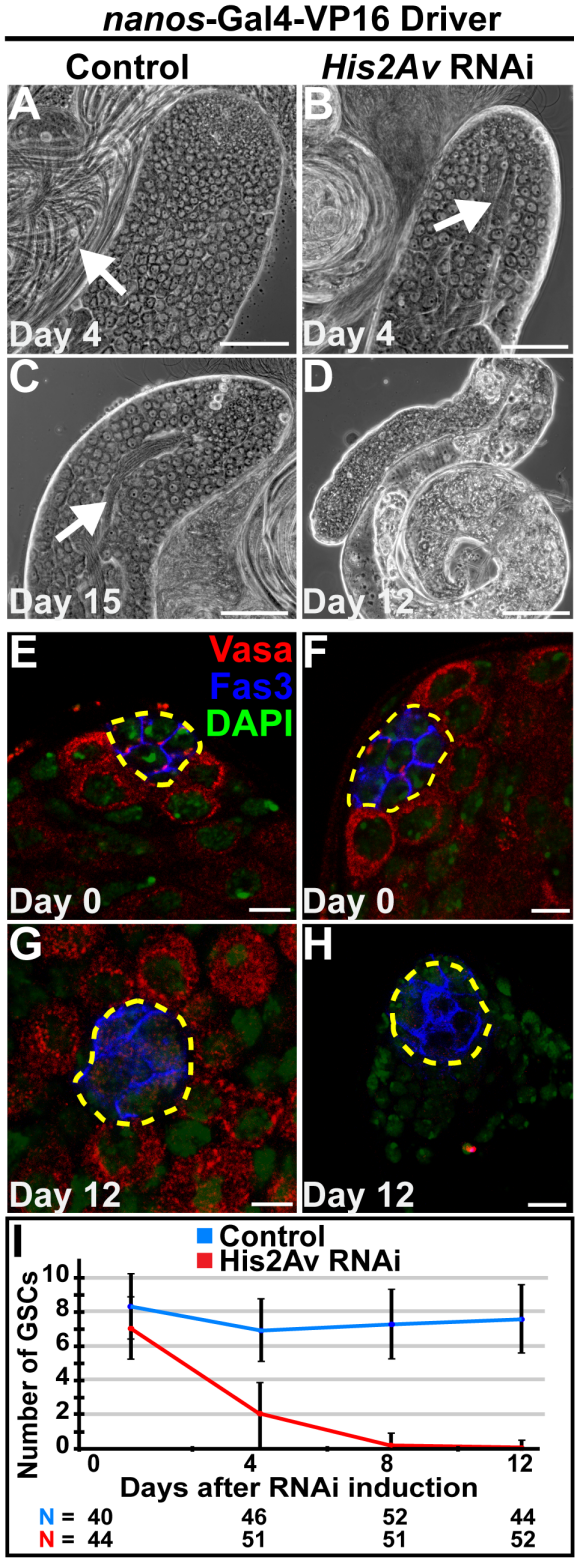
RNAi knockdown of His2Av function in early germ cells results in GSC loss. (**A–D**): Phase images of testes with RNAi knockdown of *His2Av* in GSCs and early germ cells using the NG4VP16 driver (B, D) and sibling control (A, C) at day 4 (A, B), day 12 (D) and day 15 (C) after RNAi induction. Arrows point to elongated spermatids. Scale Bars: 50 µm (**E–H**): Apical tips of testes with RNAi knockdown of *His2Av* in the germline (F, H) and sibling control (E, G) at day 0 (E, F) and day 12 (G, H) after RNAi induction and immunostained with anti-Vasa (red), anti-Fas3 (blue), and DAPI (green); hub (yellow dashed line). Scale bars (A–E): 10 µm(**I**): Quantification of GSC number over days after *His2Av* RNAi induction (red line) and sibling control (blue line). Data shows average ± S.D.

Knockdown of His2Av function specifically in GSCs and early germ cells by expression of a RNAi hairpin for *His2Av* using the *nanos-GAL4-VP16* (NGVP16) driver also indicated a cell autonomous role for His2Av in GSC maintenance. By day 3 after RNAi expression, induced by shifting flies from 18°C to 30°C, His2Av protein levels in GSCs dropped considerably compared to controls (Fig. S2A, B). At day 4 after RNAi induction, visualization of testes by phase contrast microscopy revealed the presence of spermatocytes and elongated spermatids in testes expressing *His2Av* RNAi and in controls (Fig. 2A, B). By day 12, however, testes expressing *His2Av* RNAi exhibited germ cell loss and did not contain spermatocytes or elongated spermatids (Fig. 2D), while control testes at day 15 still had both cell types (Fig. 2C). Quantitation of GSC number revealed that the loss of germ cells observed 12 days after RNAi induction was due to a failure to maintain GSCs. At day 0, *His2Av* RNAi expressing and control testes had an average of 7 and 8.2 GSCs, respectively (Fig. 2E, F, I). By day 12, the number of GSCs adjacent to the hub in testes expressing *His2Av* RNAi had dropped to 0, while control testes contained an average of 7.8 GSCs per testis hub (Fig. 2G, H, I).

### *His2Av* is not required for germ cell differentiation

**Figure 3:**
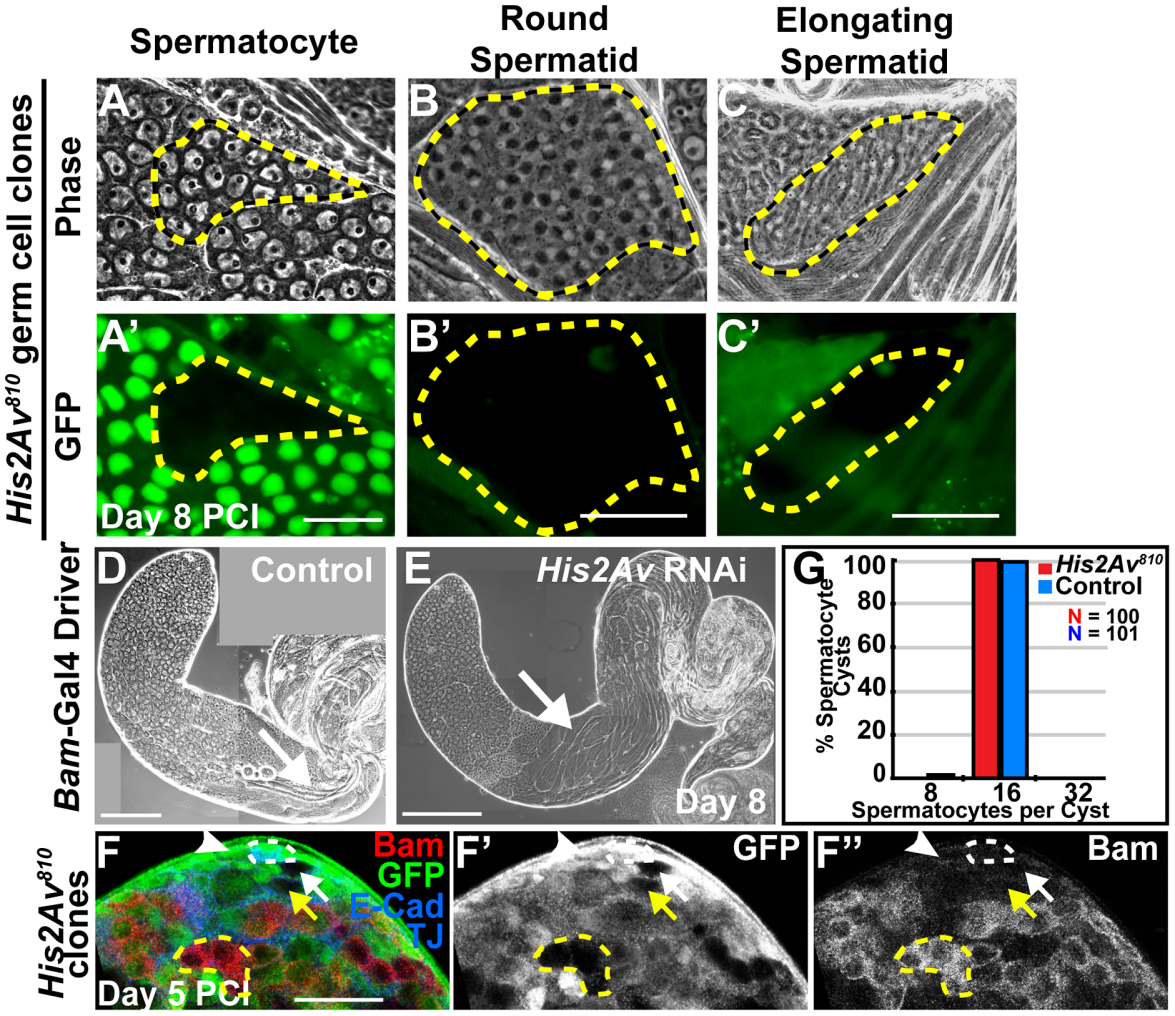
*His2Av* is not required for germ cell differentiation. (**A–C′**): Phase (A, B, C) and GFP (A′, B′, C′) images of GFP-negative *His2Av^810^* spermatocyte (A, A′), round spermatid (B, B′), and elongating spermatid (C, C′) cysts at day 8 PCI. Mutant clones (yellow dashed line). (**D, E**): Phase images of testes with RNAi knockdown of *His2Av* in spermatocytes using the Bam-Gal4 driver (E) and sibling control (D) at day 8 after RNAi induction. Arrows point to elongated spermatids. Scale Bars (A–E): 50 µm (**F–F″**): Immunostaining with anti-Bam (F, red and F″), anti-GFP (F, green and F′), anti-E-Cad and TJ (F, blue) at day 5 PCI. *His2Av^810^* mutant GSC (white arrow), *His2Av^810^* mutant gonialblast (yellow arrow), *His2Av^810^* heterozygous GSC (white arrowhead), *His2Av^810^* mutant 4-cell spermatogonial cyst (yellow dashed line) and hub (white dashed line). Scale bar: 25 µm (**G**): Number of spermatocytes per cyst of *His2Av^810^* heterozygous (blue) and mutant (red) clones counted at day 8 PCI.

In contrast to its role in GSCs, His2Av was not required cell autonomously for germ cell differentiation. Germline clones homozygous mutant for *His2Av^810^* differentiated into spermatocytes (Fig. 3A and Fig. S1A) and round and elongating spermatids (Fig. 3B, C), as observed 8 days after clone induction. Mutant onion stage round spermatids had the normal size and 1∶1 ratio of nuclei to mitochondrial derivatives, indicating successful progression through meiotic divisions (Fig. 3B). Knockdown of His2Av in late spermatogonial cysts by RNAi expressed under the control of the *bam-*Gal4 driver confirmed that His2Av function is dispensable for the differentiation program of germ cells at the later stages. His2Av protein levels were greatly reduced in spermatocytes upon expression of RNAi (Fig. S2C, D), yet spermatocytes lacking His2Av protein for 8 days after RNAi induction were still able to differentiate, undergo meiosis, and give rise to elongated spermatids (Fig. 3D, E).

Although *His2Av* mutant GSCs were lost to differentiation, they did not appear to do so by accumulating Bam protein earlier than their heterozygous counterparts. The accumulation of Bam protein in transit-amplifying spermatogonial cells stops proliferation and initiates differentiation to spermatocytes [16]. Immunostaining for Bam protein 5 days PCI revealed that neither heterozygous *His2Av^810^*/+ nor homozygous *His2Av^810^* mutant GSCs or gonialblasts expressed Bam protein (Fig. 3F). Bam protein did accumulate at the correct time during the differentiation program in *His2Av* mutant cells, at the 4-cell spermatogonial stage (Fig. 3F), similar to in wild-type spermatogonial cysts. Consistent with the correct temporal accumulation of Bam protein, germ cells lacking His2Av function underwent 4 rounds of spermatogonial divisions, producing cysts with 16 spermatocytes (Fig. 3G).

### *His2Av* is required cell autonomously for CySC maintenance but not for somatic cyst cell differentiation

**Figure 4:**
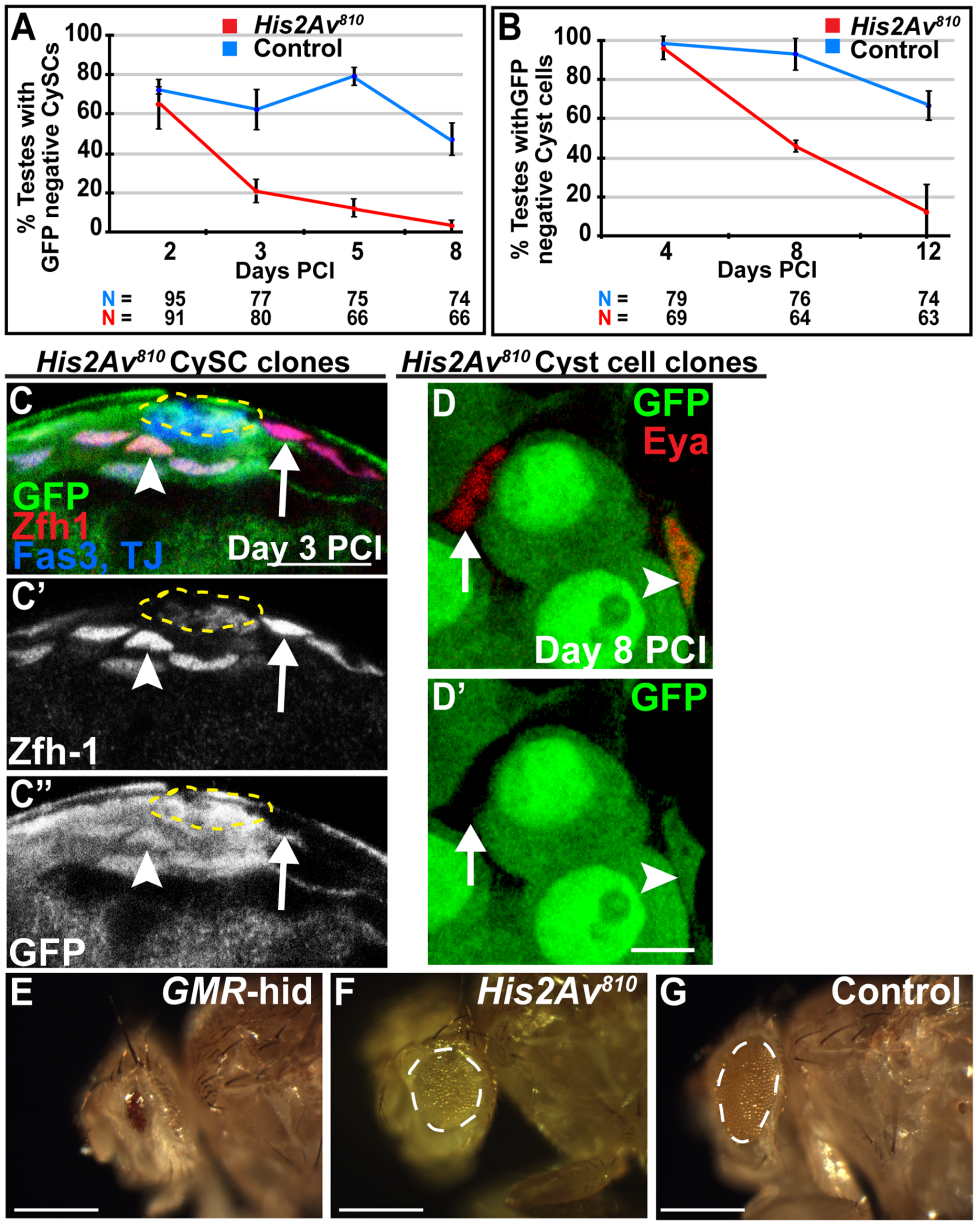
*His2Av* is required cell autonomously for CySC maintenance but not for somatic cyst cell differentiation. (**A, B**): Percentage of testes with *His2Av^810^* mutant (red line) or FRT 82B control (blue line) CySCs (A) or cyst cells (B) scored at indicated time points PCI. Data shows average ± S.D. (**C–C″**): Apical tip of testes 3 days PCI immunostained with anti-GFP (C, green and C″), anti-Zfh-1 (C, red and C′), and anti-Fas3 and TJ (blue). *His2Av^810^* mutant CySCs (arrow), *His2Av^810^* heterozygous CySCs (arrowhead) and hub (yellow dashed line). Scale bar: 12.5 µm (**D, D′**): Testes from 8 day PCI immunostained with anti-GFP (green) and anti-Eya (red). *His2Av^810^* mutant (arrow) and heterozygous (arrowhead) cyst cells. Scale bar: 10 µm (**E–G**): Analysis of *Drosophila* eyes derived from heterozygous GMR-*hid/+* (E), homozygous *His2Av^810^* (F), and homozygous FRT control (G) precursor cells.

Consistent with its expression in the cyst cell lineage, His2Av function was also required cell autonomously for CySC maintenance. Although both *His2Av^810^* mutant and control CySCs were present at comparable frequencies at day 2 PCI, by day 8 PCI almost all testes lacked *His2Av^810^* mutant CySCs, while control CySCs were maintained (Fig. 4A). Consistent with the loss of mutant CySCs, *His2Av^810^* mutant cyst cells expressing the differentiation marker Eya were also lost over time. *His2Av^810^* mutant cyst cells were observed in 100% of testes at day 4 PCI, but by day 12 PCI *His2Av^810^* mutant Eya-positive cyst cells were almost entirely absent (Fig. 4B). The His2Av-mRFP transgene rescued the loss of *His2Av^810^* homozygous mutant CySCs, suggesting that the failure to maintain CySCs was due to loss of His2Av function. At day 2 PCI, an average of 43.2% (n = 32) of testes contained *His2Av^810^* mutant CySCs, while under the same conditions, 67.5% (n = 23) testes from sibling males carrying the His2Av-mRFP transgene contained *His2Av* mutant CySCs (data not shown). The percentage of testes containing *His2Av^810^* mutant CySCs at day 8 PCI dropped to 3% (n = 26), while 42.1% (n = 31) of testes from males carrying the His2Av-mRFP transgene contained marked CySCs.

The failure to maintain CySCs was not due to downregulation of the transcriptional repressor Zinc-finger homology-1 (Zfh-1), which is expressed in CySCs and is required for CySC maintenance [17]. At day 3 PCI, when only 20% of testes scored had homozygous mutant CySCs, Zfh-1 expression in *His2Av^810^* homozygous mutant CySCs was comparable to that in neighboring wild-type CySCs (Fig. 4C). As in the germ line, His2Av function was not required for cyst cell differentiation. Cyst cells lacking His2Av function differentiated successfully at least to the stage at which they express the differentiation marker Eya and are associated with differentiating germ cells (Fig. 4D).

In addition to the survival and differentiation of germ cells and cyst cells lacking His2Av, the classic eye test revealed that His2Av function might be dispensable for cell survival in the eye tissue. Eyes composed exclusively of cells lacking His2Av function were generated using the EGUF/hid system [18]. When mitotic recombination was not induced, eye precursor cells expressed the *GMR-hid* transgene and failed to develop, resulting in adult flies with tiny eyes (Fig. 4E). In contrast, when clones were induced, cells lacking His2Av function produced eyes (Fig. 4F), although they appeared slightly smaller and rougher compared to controls (Fig. 4G), suggesting that His2Av might contribute to proper cell proliferation and/or differentiation in this tissue. Together, the results from clonal and RNAi analysis in the germline, somatic cyst, and eye cell lineages suggest that in the absence of DNA damaging agents, *Drosophila* His2Av function is required for adult stem cell maintenance but not for cell survival or differentiation.

### Loss of His2Av function did not cause defects in STAT-dependent GSC characteristics

**Figure 5:**
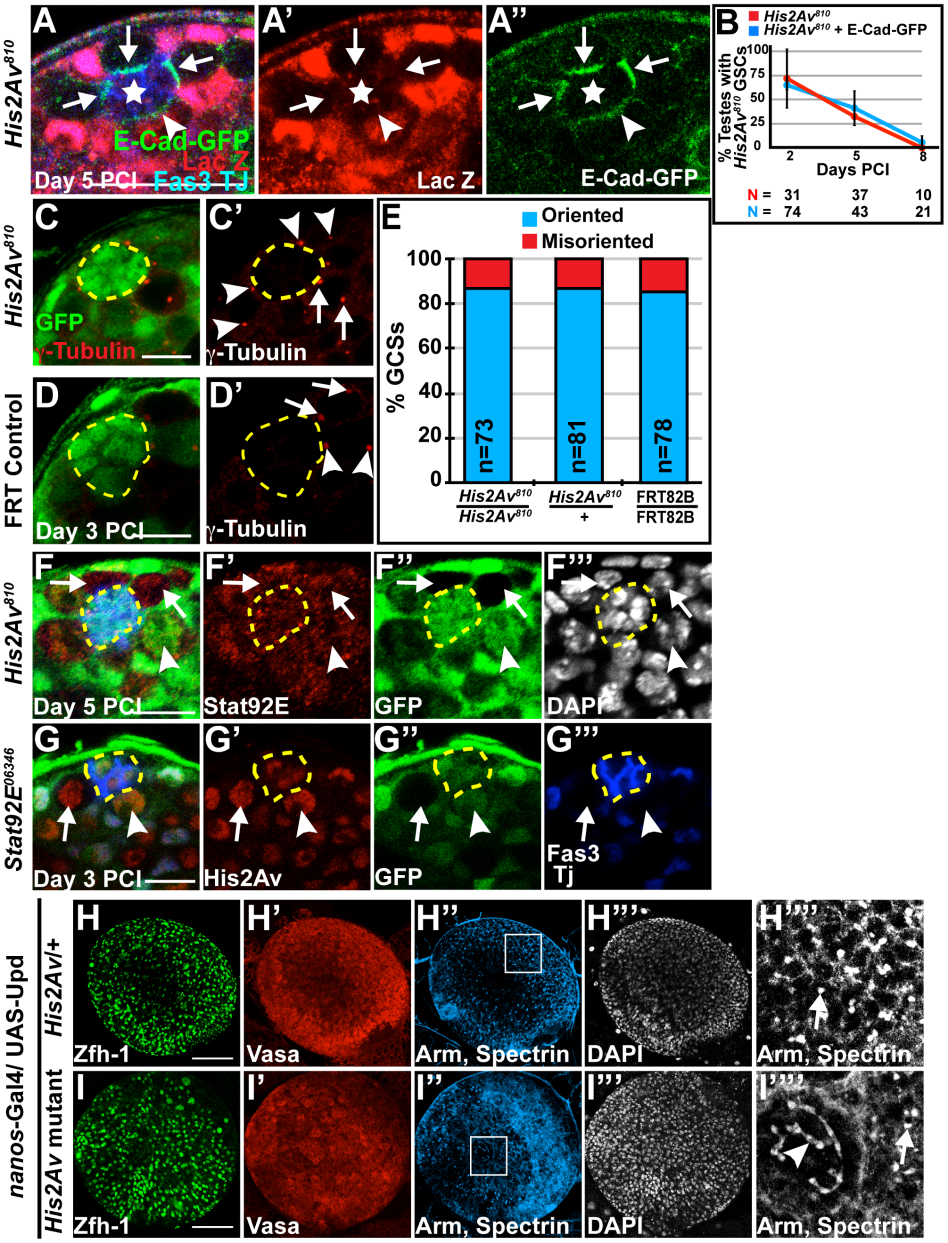
Loss of His2Av function did not cause defects in STAT dependent GSC characteristics. (**A–A″**): *His2Av^810^*/+ testes at 5 days PCI and expressing E-Cadherin-GFP under the control of the *nanos*-Gal4 driver immunostained with anti-GFP (green), anti-β-galactosidase (LacZ, red) and anti-Fas3 and TJ (blue). *His2Av^810^* mutant GSCs (arrows), *His2Av^810^* heterozygous GSC (arrowhead) and hub (star). Scale bar: 25 µm (**B**): Percentage of testes with *His2Av^810^* (red) or *His2Av^810^*, UAS-E-Cad-GFP (blue) GSCs scored at indicated times after clonal induction. Data shows average ± S.D.(**C–D′**): Immunostaining of testes 3 days after clonal induction with anti-©-tubulin (red) and anti-GFP (green) in *His2Av^810^* (C–C′) and FRT control (D–D′) GSCs. Centrosomes of GFP negative GSCs (arrows), heterozygous GFP-positive GSCs (arrowheads) and hub (yellow dashed line). Scale bars: 10 µm (**E**): Percentage of oriented (blue) and misoriented (red) centrosomes in *His2Av^810^* mutant, *His2Av^810^* heterozygous, and FRT 82B control GSCs. (**F–G′″**): Immunostaining of *His2Av^810^*/+ testes (F–F′″) 5 days PCI with anti-GFP (green), anti-STAT92E (red) and anti-Fas3 and TJ (blue) and *STAT92E^06346^*/+ testes (G–G′″) 3 days PCI with anti-GFP (green), anti-His2Av (red) and anti-Fas3 and TJ (blue). DAPI (F′″) in white. *His2Av^810^* (F–F′″) and S*TAT92E^06346^* (G–G′″) mutant GSCs (arrows), *His2Av^810^* (F–F′″) and S*TAT92E^06346^* (G–G′″) heterozygous GSCs (arrowhead) and hub (yellow dashed line). Scale bar: 12.5 µm (F) and 10 µm (G). (**H–I″″**): Larval testes expressing Upd under the *nanos*-Gal4 driver and either heterozygous for *His2Av* (H–H″″) or mutant for *His2Av* (I–I″″) immunostained with anti-Zfh-1 (H, I, green), anti-Vasa (H′, I′, red), anti-Arm and anti-spectrin (H″, I″, blue and H″″, I″″) and counterstained with DAPI (H′″, I′″). H″″ and I″″ are magnifications of the regions enclosed in H″ and I″, respectively. Dot fusomes (arrows) and branched spectrosomes (arrowhead). Scale bar: 100 µm.

Analysis of *His2Av* mutant GSCs revealed that His2Av function was not required to maintain three previously defined STAT-dependent characteristics of GSCs: 1) attachment to the hub through E-cadherin mediated adherens junctions, 2) oriented cell division [19], and 3) upregulation of STAT92E protein in response to Unpaired (Upd) signaling from the hub. *His2Av* mutant GSCs localized E-Cadherin-GFP (E-Cad-GFP), expressed in GSCs by the *nanos*-Gal4 driver and detected 5 days PCI, to the hub-GSC interface similar to neighboring heterozygous GSCs (Fig. 5A) and as previously shown [12]. The expression of E-Cad-GFP in GSCs did not result in an increase in *His2Av* mutant GSC maintenance; *His2Av^810^* mutant GSCs in testes from sibling males either expressing or lacking the expression of E-Cad-GFP were lost at the same rate (Fig. 5B). His2Av also did not appear to be required for the stereotypical orientation of centrosomes in GSCs that sets up the mitotic spindle orientation and the subsequent asymmetric outcome of GSC division [12]. Analysis of testes 3 days after induction of *His2Av^810^* clones revealed that in GSCs with two centrosomes, one centrosome was found adjacent to the hub-GSC interface in 86.3% of *His2Av^810^* mutant GSCs, similar to neighboring heterozygous *His2Av^810^/+* GSCs (86.42%) and FRT control GSCs (84.62%) (Fig. 5C–E). Loss of His2Av function did not substantially alter the accumulation of STAT92E, an indicator of JAK-STAT activity [20], in GSCs. *His2Av^810^* mutant GSCs remaining adjacent to the hub 5 days PCI had STAT92E protein levels comparable to neighboring heterozygous GSCs (Fig. 5F), suggesting that loss of GSCs in *His2Av* mutants is not due to failure to express STAT92E. Conversely, GSCs homozygous mutant for either *Stat92E^06346^* (Fig. 5G) or *Stat92E^jc46^* (data not shown) expressed His2Av protein at levels comparable to neighboring heterozygous GSCs, suggesting that His2Av expression in GSCs was not dependent on STAT92E function.

Loss of His2Av function did not suppress the overproliferation of CySC-like and GSC-like cells in testes with forced activation of the JAK-STAT pathway. When the Upd ligand was expressed ectopically in early germ cells under the control of the *nanos*-Gal4 driver, larval testes heterozygous for *His2Av* had an abundance of small Vasa-positive GSC-like cells with dot spectrosomes and Zfh-1 positive CySC-like cells (Fig. 5H). Under the same conditions, testes from sibling *nos*-Gal4/UAS-*Upd*; *His2Av^810^*/Df(3R) BSC524 larvae also exhibited an abundance of GSC-like and CySC-like cells (Fig. 5I), although there were subtle signs of differentiating germ cells. In the absence of His2Av function, 16 out of 37 (43.24%) *nos-*Gal4; UAS-Upd larval testes had a few germ cell cysts containing branched fusomes (Fig. 5I″″). In the same experiment, only 1 out of 37 (2.7%) testes from *nos*-Gal4/UAS-Upd; *His2Av*/+ larvae exhibited branched fusomes. Thus, in the absence of His2Av function, a small population of *His2Av* mutant germ cells appears to initiate the differentiation program even under conditions of high JAK-STAT activation.

### The chromatin remodeling factor Domino appears to function in the His2Av pathway to maintain GSCs and CySCs

**Figure 6:**
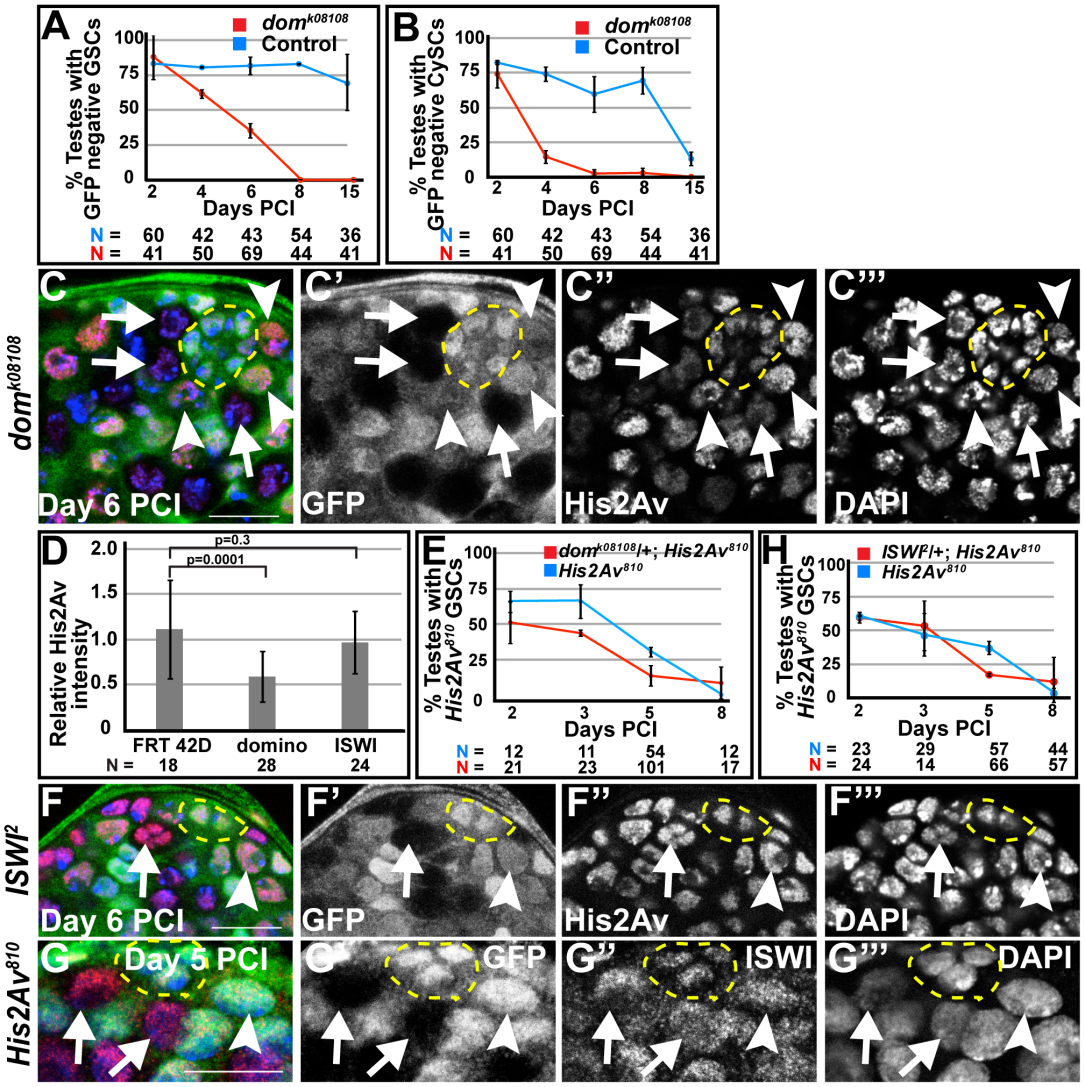
*Domino* function is required for the association of His2Av with chromatin. (**A, B**): Percentage of testes with *dom^k08108^* mutant (red) or FRT 42D control (blue) GSCs (A) and CySCs (B) scored at indicated times after clonal induction. Data shows average ± S.D. (**C–C′″**): Apical tip of *dom^k08108^*/+ testes at day 6 PCI immunostained with anti-GFP (C, green and C′) and anti-His2Av (C, red and C″) and counterstained with DAPI (blue, C and C′″). *dom^k08108^* mutant GSCs (arrows), *dom^k08108^* heterozygous GSCs (arrowheads) and hub (yellow dashed line). Scale bar: 12.5 µm (**D**): Average ratio of His2Av intensity per unit area in GSC homozygous for FRT 42D, *dom^k08108^* or *ISWI^2^* to neighboring GSCs heterozygous for FRT 42D, *dom^k08108^* or *ISWI^2^*, respectively. Data shows average ± S.D. P-values of Student's t-test (2-tailed) are shown. (**E**): Percentage of testes with *His2Av^810^* (blue) or *His2Av^810^* ;*dom^k08108^*/+ (red) GSCs scored at indicated times after clonal induction. Data shows average ± S.D. (**F–G′″**): Apical tip of *ISWI^2^*/+ (F–F′″) testes at day 6 PCI or *His2Av^810^*/+ (G–G′″) testes at day 5 PCI immunostained with anti-GFP (F, G, green and F′, G′) and anti-His2Av (F, red and F″) or anti-ISWI (G, red and G″) and counterstained with DAPI (blue, F, G and F′″, G′″). *ISWI^2^* (F–F′″) or *His2Av^810^* (G–G′″) mutant GSCs (arrows), *ISWI^2^* (F–F′″) or *His2Av^810^* (G–G′″) heterozygous GSCs (arrowheads) and hub (yellow dashed line). Scale bars: 12.5 µm (**H**): Percentage of testes with *His2Av^810^* (blue) or *His2Av^810^* ;*ISWI^2^*/+ (red) GSCs scored at indicated times after clonal induction. Data shows average ± S.D.

Clonal analysis suggested that the chromatin remodeling factor Domino, the homolog of yeast Swr1 [21], which exchanges His2A variant for His2A in yeast [22], [23], is required for stem cell maintenance. In the *Drosophila* testis, when negatively marked clones of *dom^k08108^* were generated using the FLP/FRT system, the percentage of testes carrying marked *dom^k08108^* homozygous GSCs or CySCs was indistinguishable from the control at day 2 PCI (Fig. 6A, B). However, the percentage of testes carrying marked *dom^k08108^* homozygous GSCs steadily decreased over time after clonal induction and dropped to zero by day 8 (Fig. 6A). Similarly, the percentage of testes with *dom^k08108^* mutant CySCs dropped from 74% at day 2, to 14.5% at day 4, to 0% at day 15 (Fig. 6B). Immunostaining analysis revealed that Domino function might be required for the localization of His2Av to chromatin in GSCs, similar to the function of the corresponding Swr1 complex in yeast. At day 6 PCI, nuclei in GSCs lacking Domino function had reduced levels of His2Av protein compared to control GSCs (Fig. 6C–C′″). Quantification of His2Av immunofluorescence intensity revealed that the loss of *domino* function reduced His2Av protein levels in GSCs by an average of 2-fold. The average ratio of His2Av immunostaining per unit area in GSCs that were homozygous for *dom^k08108^* compared to His2Av immunostaining per unit area in GSCs heterozygous for *dom^k08108^* within a testis (n = 28 testes) was 0.56. In contrast, in FRT 42D control (n = 18 testes), the ratio of His2Av immunostaining per unit area in GFP negative to GFP positive GSCs was 1.12 (Fig. 6D). Consistent with a role for Domino in His2Av incorporation and function in GSC maintenance, the loss of *His2Av^810^* mutant GSC clones increased in a *dom^k08108^*/+ genetic background (Fig. 6E). At day 2 PCI, 65.5% of testes contained *His2Av^810^* homozygous mutant GSC clones, while under the same conditions, only 51.1% of testes from sibling males carrying the *dom* allele had marked GSC clones, possibly due to reduced incorporation of His2Av into chromatin before clonal induction. Under the same conditions at day 2 PCI, an average of 92% of testes from both *dom^k08108^*/+; *His2Av^810^* and sibling *His2Av^810^* males had spermatocyte clones, suggesting that clonal induction occurred at the same rate in both genetic backgrounds (data not shown). The percentage of testes with marked *His2Av* mutant GSCs was also lower at days 3 and 5 PCI in males with the *dom^k08108^*/+ allele compared to sibling males without the *dom* allele.

In contrast to the function of Domino, the chromatin remodeling factor ISWI, which also functions in GSC and CySC maintenance [24], did not appear to be required for the localization of His2Av to chromatin in GSCs. Immunostaining for His2Av protein in testes containing *ISWI^2^* homozygous mutant GSCs 6 days PCI revealed that the levels of His2Av protein in *ISWI* mutant GSCs were comparable to neighboring *ISWI^2^*/+ GSCs (Fig. 6F). The average ratio of His2Av immunostaining intensity per unit area for GSCs homozygous mutant for *ISWI^2^* to neighboring *ISWI^2^*/+ GSCs within the same testis (n = 24 testes) was 0.96 (Fig. 6D). Similarly, ISWI protein levels in the nuclei of *His2Av^810^* mutant GSCs were comparable to that in heterozygous GSCs (Fig. 6G), suggesting that His2Av might not be required to recruit or maintain ISWI on chromatin. *ISWI* did not exhibit a strong genetic interaction with *His2Av* to maintain GSCs in the adult testes. The percentage of testes with marked *His2Av^810^* mutant GSC clones in *ISIW^2^*/+; *His2Av^810^* testes was comparable to that in testes lacking the *ISWI* allele at days 2, 3, and 8 PCI, only falling slightly at day 5 PCI (Fig. 6H).

**Figure 7:**
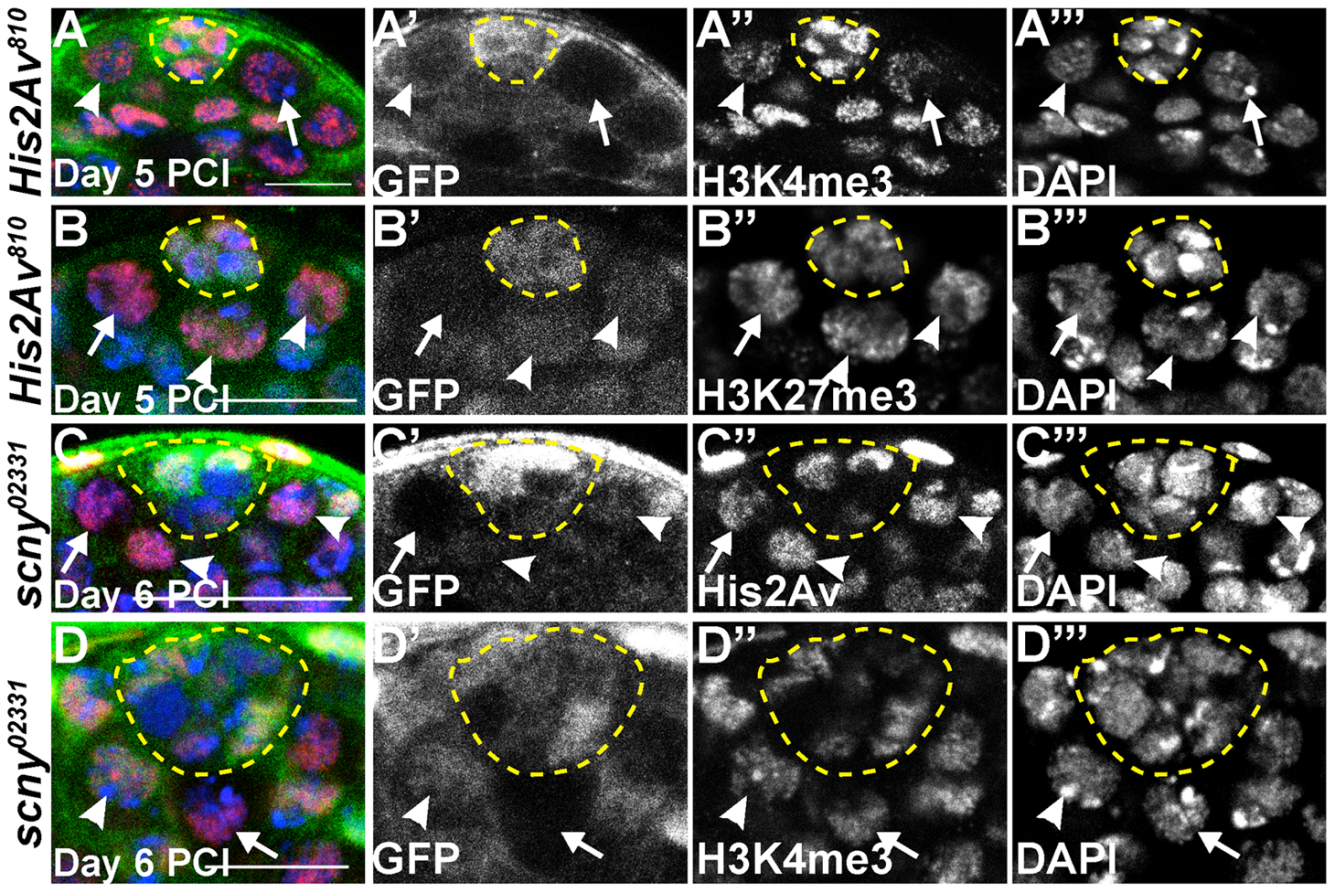
*His2Av* mutant GSCs do not exhibit dramatic changes in epigenetic markers of transcriptional state. (**A–B′″**): *His2Av^810^*/+ testes 5 days PCI immunostained with anti- GFP (A, B, green and A′, B′), anti-H3K4me3 (A, red and A″) or anti-H3K27me3 (B, red and B″) and counterstained with DAPI (A, B, blue and A′″, B′″). *His2Av^810^* mutant GSCs (arrows), *His2Av^810^* heterozygous GSCs (arrowhead) and hub (yellow dashed line). Scale bar: 10 µm (A) and 12.5 µm (B). (**C–D′″**): *scny^02331^*/+ testes 6 days PCI immunostained with anti- GFP (green, C, D and C′, D′), anti-His2Av (red, C and C″) or anti-H3K4me3 (red, D and D″) and counterstained with DAPI (blue, C, D and C′″, D′″). *scny^02331^* mutant GSCs (arrows), *scny^02331^* heterozygous GSCs (arrowheads) and hub (yellow dashed line). Scale bar: 25 µm (C) and 12.5 µm (D).

Loss of His2Av function did not globally alter levels of the epigenetic marks associated with transcriptional state in GSCs. Immunostaining with antibodies that recognize H3K4 tri-methyl (H3K4me3) (Fig. 7A), mostly associated with transcriptionally active/poised chromatin regions, and H3K27 tri-methyl (H3K27me3) (Fig. 7B), mostly associated with transcriptionally inactive regions of chromatin [1] on testes with *His2Av^810^* mutant GSCs 5 days PCI revealed that the levels of these epigenetic marks were comparable in mutant and heterozygous GSCs. Likewise, the protein levels of Scrawny (Scny), a histone H2B deubiquitinase required to prevent premature expression of differentiation genes in adult stem cells [25], were also not altered in *His2Av* mutant GSCs (data not shown). Furthermore, His2Av protein levels scored 6 days PCI were unaltered in GSCs homozygous mutant for *scny^02331^* (Fig. 7C) or s*cny^e00340^* (data not shown) compared to neighboring *scny*/+ heterozygous GSCs. Although *scny* mutant follicle cells in the *Drosophila* ovary exhibit elevated levels of H3K4me3 [25], male GSCs homozygous mutant for either *scny^02331^* (Fig. 7D) or s*cny^e00340^* (data not shown) did not exhibit changes in H3K4me3 levels compared to neighboring heterozygous GSCs. These data suggest that loss of His2Av or Scny function was not associated with dramatic changes in transcription in the testis, at least when assayed at a global level by immunostaining for histone marks.

## Discussion

Our results reveal that the histone variant His2Av is required cell autonomously for maintenance of two different adult stem cell types, GSCs and CySCs, in the *Drosophila* male gonad, but not for the differentiation of the progeny in these two stem cell lineages. The specific requirement for His2Av for adult stem cell maintenance suggests that His2Av may play critical role(s) in the mechanisms that maintain the ability of adult stem cells to self-renew rather than differentiate. His2Av function has been implicated in both transcriptional repression and transcriptional activation. His2Av could maintain adult stem cells by either favoring repression of pro-differentiation genes and/or activation of genes necessary for stem cell identity and function. In yeast, H2A.Z occupies transcriptionally inactive genes and intergenic regions [26], while in *Drosophila*, His2Av is required for the establishment of heterochromatin and transcriptional repression [27]. Conversely, studies indicate that in *Drosophila*, yeast, and chicken, His2Av is enriched at nucleosomes downstream of the transcription start site of active or poised genes [28], [29], [30]. Nucleosomes and histone dimers containing H2A.Z appear to be less stable than nucleosomes containing the canonical histone H2A [31], [32], [33]. This lower stability may favor a more open chromatin, giving transcriptional activators or repressors better access to the DNA. Consistent with this model, a recent study showed that H2A.Z promotes self-renewal and pluripotency of murine embryonic stem cells (ESCs) by facilitating the binding of Oct4 to its target genes and the Polycomb repressive complex 2 to differentiation genes [34]. However, in ESCs, unlike in *Drosophila* male GSCs and CySCs, His2A.Z function was also required for the expression of differentiation genes when ESCs were grown under conditions that induce differentiation [34], [35].

We propose that the requirement of His2Av for adult stem cell maintenance, but not for differentiation, may reflect a subtle role for His2Av in maintaining expression of genes required for self-renewal versus differentiation. Adult stem cells lie at the cusp of two alternate fate choices, self-renewal and differentiation; the progeny of stem cell division are maintained in a state where they can execute either self-renewal or differentiation programs depending on local cues. The requirement for this balanced, bi-potential state may make adult stem cells more sensitive to the small alterations in the relative levels of key transcripts associated with the loss of His2Av function, tilting the balance from stem cell maintenance to onset of differentiation. Consistent with the model that His2Av may alter transcriptional levels subtly, H2A.Z was shown to be required to fine-tune the transcriptional state of *hsp70* and a wide variety of other genes in response to temperature changes in *Arabidopsis* [36], [37].

The ATP-dependent chromatin-remodeling factor Domino is required for GSC and CySC maintenance in the male germline, as previously shown for somatic follicle stem cells in the female gonad [38]. The yeast Swr1 complex containing the homolog of *Drosophila* Domino exchanges His2A with Htz1 (the yeast His2A variant) [22], [23], [39] and in *Drosophila*, Domino- containing Tip60 chromatin remodeling complex has been shown to exchange phospho-His2Av with unmodified His2Av in in vitro assays [40]. Our studies indicate that Domino function is required in vivo in GSCs for the incorporation of His2Av into chromatin. Nuclei of *domino* mutant GSCs had lowered but still detectable levels of His2Av protein, possibly due to the weak *domino* allele used in this study. Alternatively, incorporation of His2Av in some regions of the chromatin may occur independently of Domino function, as has been reported in yeast, in which stress-responsive genes exhibit Swr1-independent incorporation of Htz in the coding region [41]. Although ISWI, like His2Av, is required for GSC and CySC maintenance in the male germline [24], these proteins may function in parallel pathways to maintain adult stem cells in the testis. The ISWI containing nucleosome-remodeling factor (NURF) was shown to maintain GSCs and CySCs in the *Drosophila* testis by positively regulating the JAK-STAT signaling pathway; GSCs mutant for components of the NURF complex exhibited low levels of STAT92E protein [24]. In contrast, as discussed below, His2Av may function independently of the JAK-STAT signaling pathway.

Our results indicate that His2Av may function independently of the JAK-STAT signaling pathway to provide a chromatin environment that allows for stem cell maintenance. Expression of the His2Av and STAT92E proteins in GSCs was not dependent on each other. Our studies indicate that His2Av may not be required for expression of at least one other key STAT-dependent gene in CySCs. Activation of the JAK-STAT signaling pathway in response to the Upd signal from the hub is important for CySC maintenance, possibly in part through STAT-dependent transcription of Zfh-1 [17]. However, CySCs lacking His2Av function still expressed Zfh-1. In GSCs, activation of the JAK-STAT pathway is important for maintaining hub-GSC adhesion and for centrosome orientation [19], both of which appeared unaffected in *His2Av* mutant GSCs. Loss of His2Av function did not strongly suppress the phenotype associated with ectopic overexpression of Upd in the testis, although a few *His2Av* mutant germ cells were able to initiate differentiation, possibly due to relatively lower levels of JAK-STAT activation in these cells. Even though loss of *His2Av* normally resulted in differentiation of GSCs and CySCs, the requirement for His2Av function can be overridden by high levels of activation of the JAK-STAT pathway, possibly maintaining somatic CySCs in a stem cell like state, which may fail to provide a microenvironment for germ cells to initiate differentiation [19], [42].

## Materials and Methods

### Fly strains and husbandry

Fly stocks were raised on cornmeal/molasses medium at 25°C unless stated otherwise. Stocks are from the Bloomington Stock Center unless specified otherwise. Mutant alleles used in this study include 1) *w;FRT82B, His2Av^810^/TM6B, Tb*, carrying a 311 base pair deletion that removes the second exon of the *His2Av* gene [43], 2) *w;FRT82B, His2Av^05146^/TM3*, 3)w;; Df(3R)BSC524/*TM6b,Tb*, a deletion that encompasses the *His2Av* gene, 4) the *Stat92E* alleles, *FRT82B, Stat92E^06346^/TM3* and *FRT82B, Stat92E^J6C8^/TM3* (gift from E. Matunis), 5) *y*, *w*, ey-Flp, GMR-lacZ; FRT42D, *dom^k08108^*/*CyO*, *y^+^*, a loss of function allele (also known as *dom^1^*) with a P-element inserted at the 3′ boundary of the first exon [21] obtained from DGRC, 7) *y w*;*FRT 42D, ISWI^2^, sp*/*SM5*, *Cy*, *sp* , a null allele carrying a nonsense mutation [44], 8) the *scrawny* alleles: FRT 80B, *scny^l(3)02331^* and FRT 80B, *scny^e00340^* [25]. *w*; *His2Av-mRFP*; FRT82B, *His2Av^810^*/*TM6B, Tb* flies were used to rescue GSC and CySC loss. The His2Av-mRFP construct rescues the lethality of *His2Av^05146^* mutant [15]. The following flies 1) *hs-FLP^122^;;FRT82B, ubi-nGFP*, 2) *hs-FLP^122^;nos-GAL4;FRT82B, tub-LacZ* (gift from D. Kalderon), 3) *hs-FLP^122^;;FRT80B, ubi-nGFP*, 4) *hs-FLP^122^;FRT42D, ubi-nGFP* were used to induce marked clones in the testes. *y,w;ey-GAL4, UAS-FLP;FRT82B, GMR-hid/TM2* flies were used to induce marked clones in adult eyes. *FRT82B*, FRT80B and FRT42D were used as wild-type controls for clone induction. Other stocks used include *w,sa-GFP* [45], UAS-*Upd* [46], UAS-DEFL #6-1 (UAS-E-Cad-GFP) [47] from DGRC, *nanos*-GAL4, *UAS-Dicer2;; nanos-GAL4VP16* (*NG4VP16*) and *;; Bam-GAL4*. RNAi flies against *His2Av* (Transformant ID #110598) were obtained from the Vienna *Drosophila* RNAi Center.

A heteroallelic combination of *His2Av^810^* and Df(3R) BSC524 survives until the third instar larval stage when grown at 25°C for 2 days and then shifted to 29°C. The effects of loss of His2Av function in testes ectopically expressing Upd ligand was analysed in the third-instar larval progeny of *nanos*-GAL4; Df(3R) BSC524/TM6B,Tb and UAS-Upd; *His2Av^810^*/TM6B,Tb. Tb-positive larvae (heterozygous for either *His2Av^810^* or Df(3R) BSC524) expressing UAS-Upd under the *nanos*-Gal4 driver were used as controls

### Immunofluorescence

Testes were dissected in 1× phosphate-buffered saline (PBS) and fixed in 4% formaldehyde in PBS for 20 minutes at room temperature, washed twice for 30 minutes each in PBS with 0.3% Triton X-100 and 0.6% sodium deoxycholate. Testes were incubated overnight at 4°C in primary antibodies against Armadillo (Arm, mouse 1∶10; Developmental Studies Hybridoma Bank (DSHB)) [48], Fas3 (mouse 1∶10; DSHB) [49], α-spectrin (mouse 1∶10; DSHB) [50], Eyes absent (Eya, mouse 1∶10; DSHB) [51], E-cadherin (mouse 1∶10, DSHB) [52], Green Fluorescent protein (GFP, rabbit 1∶400–1∶1000; Invitrogen and Sheep 1∶1000, Abd-Serotec), β-Galactosidase (rabbit 1∶1000; Cappel), Histone H3 lysine 4 trimethyl (H3K4me3, rabbit 1∶200: Cell Signaling), Histone H3 lysine 27 trimethyl (H3K27me3, rabbit 1∶200: Cell Signaling), His2Av (rabbit 1∶1000; gift from R. Glaser) [53], Traffic-jam (Tj, guinea pig 1∶5000; gift from D. Godt) [54], Vasa (goat 1∶50; Santa Cruz Biotechnology), ©-tubulin (mouse 1∶50; Sigma), Zfh-1 (rabbit 1∶5000; gift from R. Lehman), STAT92E (rabbit 1∶1000; gift from E.Bach) [55], Scrawny (guinea pig 1∶200; gift from M. Buszczak) [25] and ISWI (rabbit 1∶100; gift from J.Tamkun) [56]. Secondary antibodies used were from the Alexa Fluor-conjugated series (1∶500; Molecular Probes). Samples were mounted in VECTASHIELD medium containing DAPI to visualize DNA (Vector Labs H-1200). Immunofluorescence images were obtained with a Leica SP2 Confocal Laser Scanning microscope. Phase and clonal analysis images were obtained using a Zeiss Axioskop microscope and SPOT RT3 camera by Diagnostic Instruments or CoolSNAPez camera by Photometrics. Images were processed using Adobe CS4 Photoshop and Illustrator. Comparison of intensity of His2Av staining in GSCs was performed using the ImageJ program [57]. The nuclear area in GSCs was selected based on the DAPI staining and the average intensity of His2Av immunostaining within the nucleus was measured using ImageJ. An average of immunofluorescence intensity per unit area for all GSCs homozygous (identified as GFP negative) or heterozygous (identified as GFP positive) for a given genotype was calculated for each testis. The relative level of His2Av protein was calculated as a ratio of the average immunofluorescence intensity per unit area for homozygous GSC to heterozygous GSC within each testis. Similar results were obtained when anti-His2Av intensity was normalized to the intensity for DAPI staining for each GSC.

### Clonal and RNAi analysis

Homozygous His2Av mutant clones in a heterozygous background were generated by crossing either 1) hs-FLP^122^;;FRT82B, ubi-nGFP, 2) hs-FLP^122^; FRT42D, dom^k08108^/CyO;FRT82B, ubi-nGFP, 3) hs-FLP^122^; FRT 42D, ISWI^2^, sp/CyO;FRT82B, ubi-nGFP, or 4) hs-FLP^122^;nos-GAL4;FRT82B, tub-LacZ virgin females to w;;FRT82B, w;;FRT82B, His2Av^810^/TM6B, Tb or w;; UAS-DEFL #6-1, FRT82B, His2Av^810^/TM6B, Tb [The UAS-DEFL #6-1 (UAS-E-Cad-GFP) containing chromosome was recombined to the FRT82B, His2Av^810^ chromosome] males. Homozygous dom^k08108^ or ISWI^2^ mutant clones were obtained by crossing males of the alleles to hs-FLP^122^;FRT42D, ubi-nGFP virgin females, while males of scny alleles were crossed to hs-FLP^122^;;FRT80B, ubi-nGFP males. The progeny were raised at 25°C and heat-shocked at 37°C for two hours each on two consecutive days at the pupal stage. GSCs homozygous mutant for His2Av^810^ or other alleles were identified by their lack of GFP (or β-Galactosidase), presence of the germ cell marker Vasa, and contact with the hub. Homozygous clones of CySCs generated by heat shock induced mitotic recombination were identified by their lack of GFP (or β-Galactosidase) and the germ cell marker Vasa, by the presence of Tj, a marker of the cyst cell lineage, and by their proximity to the hub.

Homozygous mutant germline clones generated in *His2Av^05146^*/+ resulted in the loss of mutant GSCs (Fig. S3A) and spermatocytes (Fig. S3B) over time after clone induction. However, this loss of marked cells was not associated with a loss of anti-His2Av staining (Fig. S3C′), and the loss of homozygous mutant germ cells was not rescued by the presence of His2Av-mRFP transgene (Fig. S3B), suggesting that a mutation other than *His2Av* on the chromosome might be responsible for GSC loss in this line.

FLP-medicated mitotic recombination was induced in eye precursor cells by crossing *y,w;ey-GAL4, UAS-FLP;FRT82B, GMR-hid/TM2* virgins to males carrying FRT 82B, *His2Av^810^* (or FRT control). Eye precursor cells carrying one copy of the dominant cell lethal transgene *GMR*-*hid* fail to survive, thereby generating eyes composed entirely of cells homozygous for *His2Av^810^* (or the FRT control).

RNAi knockdown experiments were carried out by crossing flies carrying His2Av RNAi hairpin under the UAS regulatory sequence to either *UAS-Dicer2*;;*NG4VP16* females or *Bam-GAL4*. The progeny were raised at 18°C until eclosion and transferred to and held at 30°C.

## Supporting Information

Figure S1 Loss of *His2Av^810^* mutant spermatocytes is rescued by a transgenic His2Av-mRFP line. (A–A′″): Immunostaining of *His2Av^810^* mutant germline clones (white dashed line) with anti-GFP (green), anti-His2Av (red), anti-Vasa (magenta), and DAPI (blue) 8 days PCI. (B–C′″): Testes 12 days after induction of *His2Av^810^* mutant clones in wild-type (B–B′″) or transgenic *His2Av-mRFP* (C–C′″) background. GFP (B, C), His2Av-mRFP (B′, C′), merged (B″, C″) and phase (B′″, C′″). *His2Av^810^* clones (white dashed lines). Scale bars: 10 µm.
(TIFF)

Figure S2 Efficiency of RNAi knockdown of His2Av in germ cells. (A–B′): Testes expressing RNAi of *His2Av* under the control of NGVP16 driver (A, A′) and sibling control (B, B′) 3 days post RNAi induction stained with anti-Vasa (red), anti-Fas3 (blue), and anti-His2Av (green). GSCs (arrows), cyst cell (arrowhead) and hub (yellow dashed line). (C–D′): Testes expressing RNAi of His2Av under the control of Bam-Gal4 driver (C, C′) and sibling control (D, D′) 8 days post RNAi induction stained with anti-Vasa (red), anti-TJ (blue), and anti-His2Av (green). White bracket marks the region containing spermatocytes. Scale bars: 10 µm.
(TIFF)

Figure S3 Loss of *His2Av^05146^* mutant spermatocytes is not rescued by a transgenic His2Av-mRFP line. (A): Percentage of testes with *His2Av^05146^* mutant (red line) or FRT control (blue line) GSCs scored over time after clonal induction. (B): Percentage of testes with *His2Av^05146^* mutant spermatocyte cyst with (blue line) or without (red line) a *His2Av-mRFP* rescue transgene. Data shows average ± S.D. (C–C′″): Immunostaining of *His2Av^05146^* mutant germline clone (white line) with anti-GFP (green), anti-His2Av (red), anti-Vasa (magenta), and DAPI (blue). Scale bars: 50 µm.
(TIFF)
