# Supplementary figures and images for: The Histone Variant His2Av is Required for Adult Stem Cell Maintenance in the Drosophila Testis
Source: PLoS Genet. 2013 Nov 7;9(11):e1003903. doi: 10.1371/journal.pgen.1003903 (PMC3820763; doi:10.1371/journal.pgen.1003903)

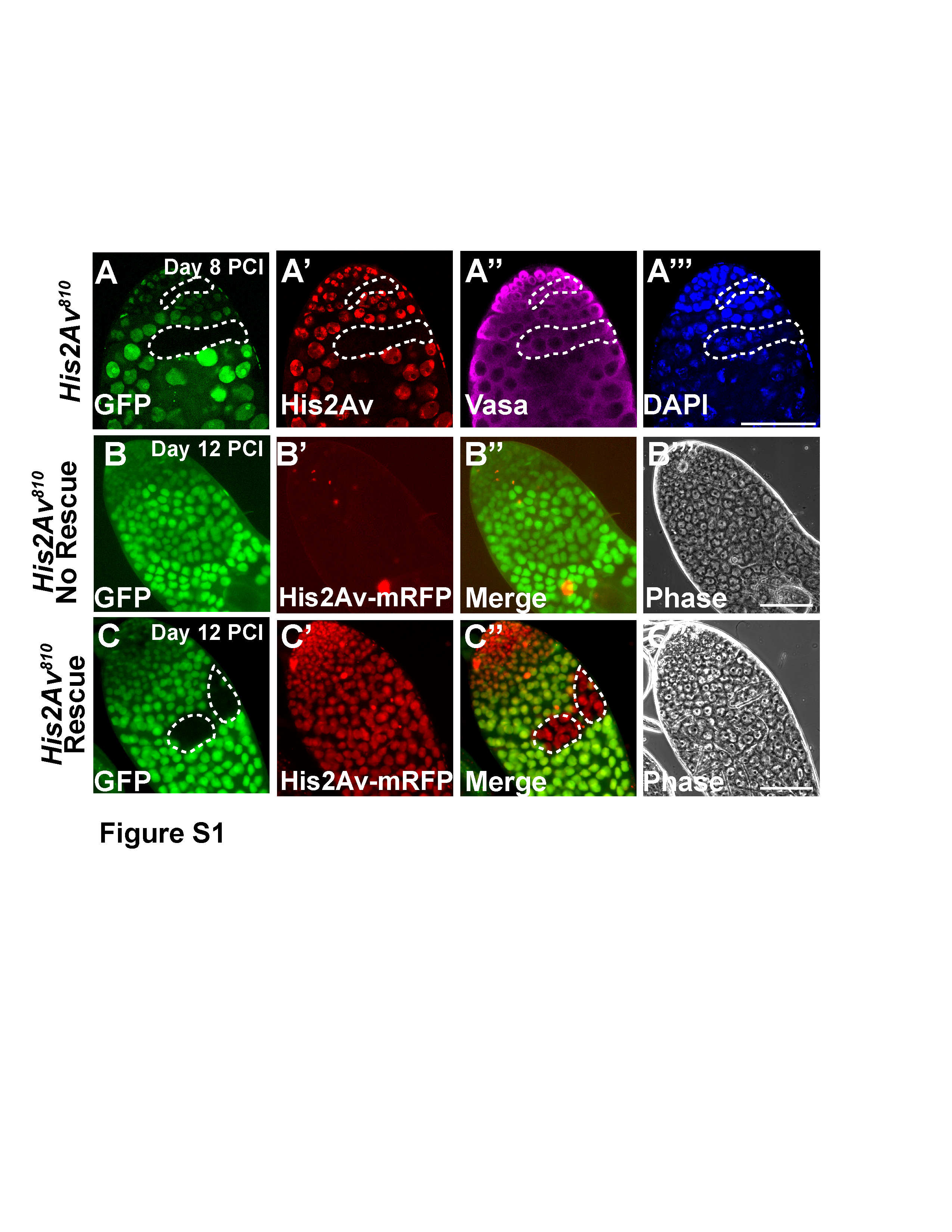

Supplement: Figure S1 — Loss of His2Av810 mutant spermatocytes is rescued by a transgenic His2Av-mRFP line. (A–A′″): Immunostaining of His2Av810 mutant germline clones (white dashed line) with anti-GFP (green), anti-His2Av (red), anti-Vasa (magenta), and DAPI (blue) 8 days PCI. (B–C′″): Testes 12 days after induction of His2Av810 mutant clones in wild-type (B–B′″) or transgenic His2Av-mRFP (C–C′″) background. GFP (B, C), His2Av-mRFP (B′, C′), merged (B″, C″) and phase (B′″, C′″). His2Av810 clones (white dashed lines). Scale bars: 10 µm. (TIFF) [file pgen.1003903.s001.tiff]

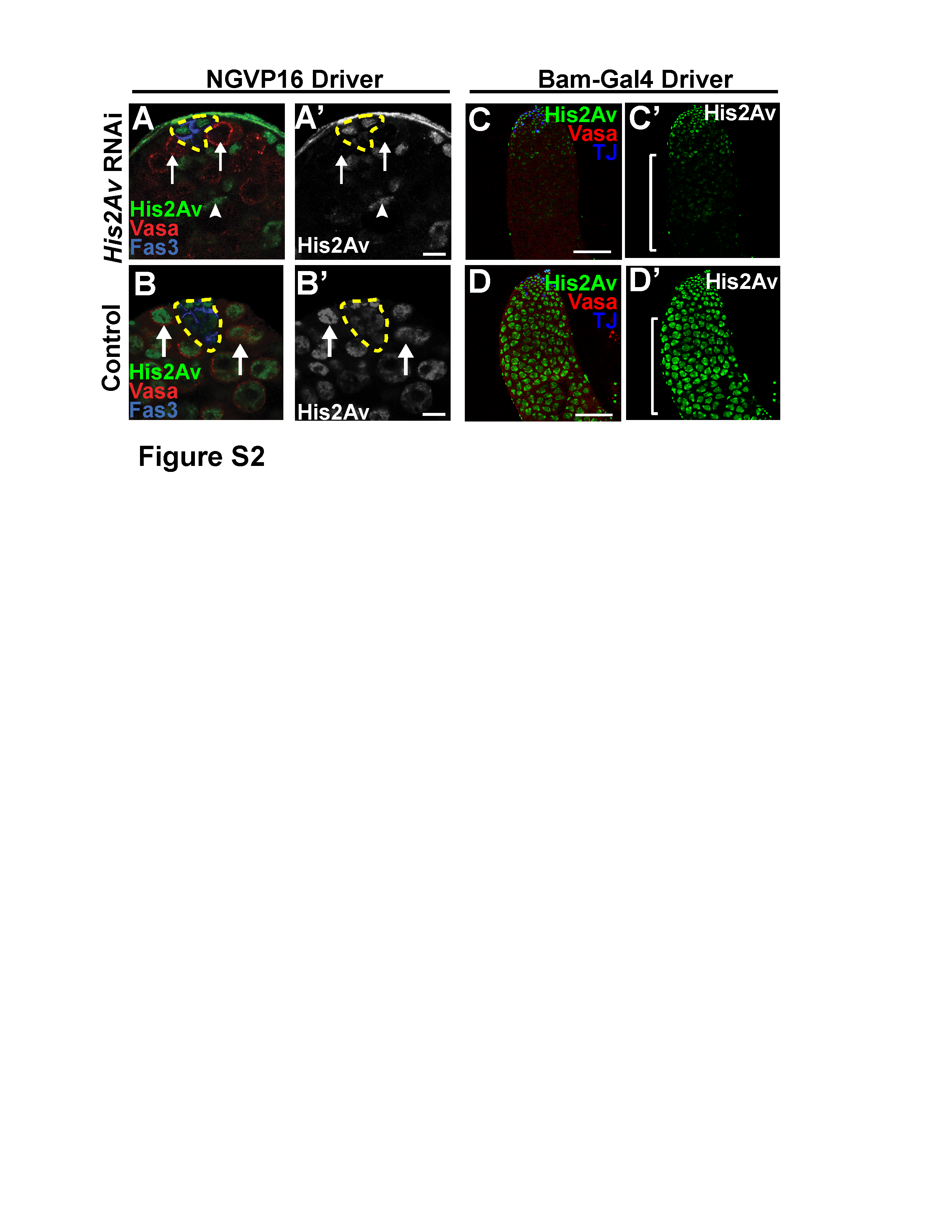

Supplement: Figure S2 — Efficiency of RNAi knockdown of His2Av in germ cells. (A–B′): Testes expressing RNAi of His2Av under the control of NGVP16 driver (A, A′) and sibling control (B, B′) 3 days post RNAi induction stained with anti-Vasa (red), anti-Fas3 (blue), and anti-His2Av (green). GSCs (arrows), cyst cell (arrowhead) and hub (yellow dashed line). (C–D′): Testes expressing RNAi of His2Av under the control of Bam-Gal4 driver (C, C′) and sibling control (D, D′) 8 days post RNAi induction stained with anti-Vasa (red), anti-TJ (blue), and anti-His2Av (green). White bracket marks the region containing spermatocytes. Scale bars: 10 µm. (TIFF) [file pgen.1003903.s002.tiff]

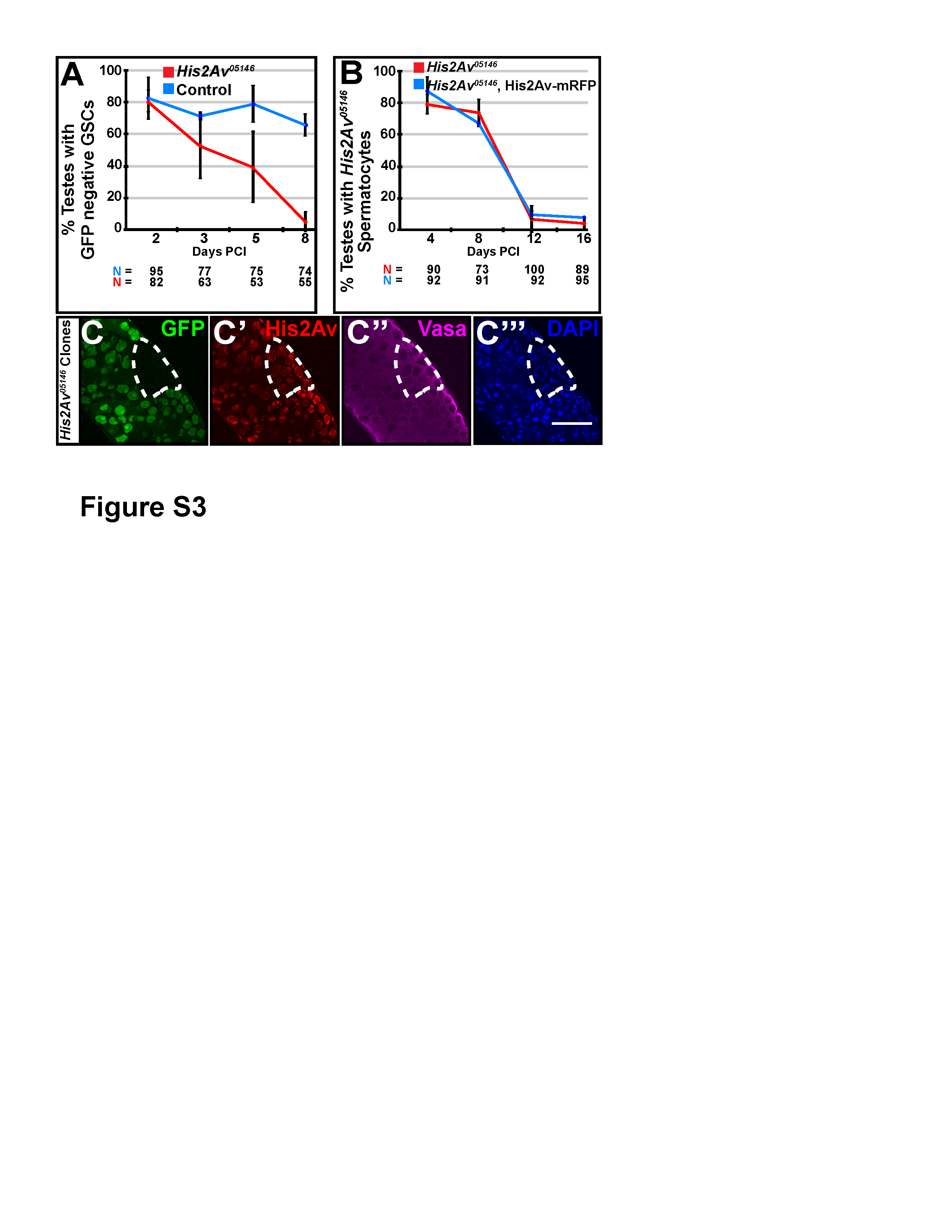

Supplement: Figure S3 — Loss of His2Av05146 mutant spermatocytes is not rescued by a transgenic His2Av-mRFP line. (A): Percentage of testes with His2Av05146 mutant (red line) or FRT control (blue line) GSCs scored over time after clonal induction. (B): Percentage of testes with His2Av05146 mutant spermatocyte cyst with (blue line) or without (red line) a His2Av-mRFP rescue transgene. Data shows average ± S.D. (C–C′″): Immunostaining of His2Av05146 mutant germline clone (white line) with anti-GFP (green), anti-His2Av (red), anti-Vasa (magenta), and DAPI (blue). Scale bars: 50 µm. (TIFF) [file pgen.1003903.s003.tiff]
